# *Vital Signs*: Colorectal Cancer Screening Test Use — United States, 2018

**DOI:** 10.15585/mmwr.mm6910a1

**Published:** 2020-03-13

**Authors:** Djenaba A. Joseph, Jessica B. King, Nicole F. Dowling, Cheryll C. Thomas, Lisa C. Richardson

**Affiliations:** 1 Division of Cancer Prevention and Control, National Center for Chronic Disease Prevention and Health Promotion, CDC.

## Abstract

**Background:**

Colorectal cancer (CRC) is the second leading cause of cancer death in the United States of cancers that affect both men and women. Despite strong evidence that screening for CRC reduces incidence and mortality, CRC screening prevalence is below the national target. This report describes current CRC screening prevalence by age, various demographic factors, and state.

**Methods:**

Data from the 2018 Behavioral Risk Factor Surveillance System survey were analyzed to estimate the percentages of adults aged 50–75 years who reported CRC screening consistent with the United States Preventive Services Task Force recommendation.

**Results:**

In 2018, 68.8% of adults were up to date with CRC screening. The percentage up to date was 79.2% among respondents aged 65–75 years and 63.3% among those aged 50–64 years. CRC screening prevalence was lowest among persons aged 50–54 years (50.0%) and increased with age. Among respondents aged 50–64 years, CRC screening prevalence was lowest among persons without health insurance (32.6%) and highest among those with reported annual household income of ≥$75,000 (70.8%). Among respondents aged 65–75 years, CRC screening prevalence was lowest among those without a regular health care provider (45.6%), and highest among those with reported annual household income ≥$75,000 (87.1%). Among states, CRC screening prevalence was highest in Massachusetts (76.5%) and lowest in Wyoming (57.8%).

**Discussion:**

CRC screening prevalence is lower among adults aged 50–64 years, although most reported having a health care provider and health insurance. Concerted efforts are needed to inform persons aged <50 years about the benefit of screening so that screening can start at age 50 years.

## Introduction

Of cancers that affect both men and women, colorectal cancer (CRC) is the second leading cause of cancer death in the United States. In 2016, 141,270 cases were diagnosed, and 52,286 persons died from the disease (*1*). The U.S. Preventive Services Task Force recommends that adults at average risk (those who do not have a personal or family history of CRC or polyps, do not have inflammatory bowel disease, or a history of genetic syndromes associated with CRC) aged 50–75 years be screened for CRC by any of six available tests: 1) fecal occult blood test (FOBT), 2) fecal immunochemical test (FIT), 3) multitarget stool DNA (FIT-DNA), 4) computed tomographic colonography (CTC), 5) sigmoidoscopy, or 6) colonoscopy (*2*). Strong evidence exists that screening for CRC reduces incidence and mortality (*2*). Both CRC incidence and mortality have declined steadily over the past 30 years; the decline is attributable in part to the increasing percentage of adults aged 50–75 years who are up to date with CRC screening (i.e., have completed a CRC screening test within the recommended time interval) (*3*,*4*). Despite steady gains, the prevalence of CRC screening is lower than the stated national Healthy People 2020 target of 70.5%, and not all populations have achieved equivalent gains in CRC screening (*5*). This report describes current CRC screening among U.S. adults aged 50–75 years, by demographic characteristics and state.

## Methods

The Behavioral Risk Factor Surveillance System (BRFSS) is an annual, state-based, random-digit–dialed telephone survey of the civilian, noninstitutionalized adult population aged ≥18 years that collects information on health risk behaviors, preventive health practices, and health care access in the United States. The median response rate for the 2018 BRFSS combined landline and cellular phone survey was 49.9% (*6*). All states and the District of Columbia asked BRFSS respondents aged ≥50 years a series of questions about their CRC screening status.* Among 222,490 respondents aged 50–75 years, 16,127 (7.2%) declined to answer, had a missing answer, or answered “don't know/not sure” and were excluded from the analysis. Screening status (up to date with CRC screening^†^) was analyzed by age groups, various demographic characteristics, and state. Data were weighted to the age, sex, and racial/ethnic distribution of each state's adult population using intercensal estimates and were age-standardized to the 2018 BRFSS population. Chi-squared tests were used to evaluate significant (p<0.005) differences in screening compliance by age group (50–64 years and 65–75 years). A test for trend was used to evaluate a significant (p<0.005) relationship between age and up-to-date screening status. SAS-callable SUDAAN (version 9.4: RTI International) was used to analyze all data.

## Results

In 2018, 68.8% of respondents reported they were up to date with CRC screening, including 79.2% of respondents aged 65–75 years and 63.3% of respondents aged 50–64 years (Table 1). Among all demographic groups studied, a significantly higher percentage of respondents aged 65–75 years reported being up to date with CRC screening than did respondents aged 50–64 years (p<0.005). The difference in the percentage of respondents who were up to date between those aged 65–75 years and those aged 50–64 years was largest (23.1 percentage points) among those without health insurance and smallest (11.1 percentage points) among respondents who identified as non-Hispanic other/multiracial. The percentage of respondents who were up to date was lowest among those aged 50–54 years (50.0%) and highest among those aged 70–75 years (81.3%); increasing age was significantly associated with an increasing percentage of persons who were up to date (p<0.005) (Figure).

**TABLE 1:** Percentage of respondents aged 50–75 years who reported being up to date* with colorectal cancer screening, by age group and selected characteristics — Behavioral Risk Factor Surveillance System (BRFSS), United States, 2018^†^

Characteristic	Age group (yrs)		
	All (50–75)	50–64	65–75^§^
	% (95% CI)	% (95% CI)	% (95% CI)
**Total**	**68.8 (68.3–69.3)**	**63.3 (62.7–63.9)**	**79.2 (78.5–79.8)**
**Sex**			
Men	67.0 (66.3–67.7)	61.1 (60.2–62.0)	78.2 (77.1–79.2)
Women	70.5 (69.9–71.2)	65.4 (64.5–66.2)	80.1 (79.2–80.9)
**Race/Ethnicity**			
White, non-Hispanic	71.0 (70.6–71.5)	65.7 (65.1–66.3)	80.7 (80.1–81.2)
Black, non-Hispanic	70.0 (68.5–71.5)	65.1 (63.2–66.9)	79.7 (76.8–82.3)
Asian/Pacific Islander, non-Hispanic	64.8 (60.7–68.7)	59.1 (54.0–64.0)	76.4 (69.8–81.9)
AI/AN, non-Hispanic	62.1 (58.6–65.5)	55.1 (50.5–59.6)	76.6 (71.7–80.8)
Other/Multiracial, non-Hispanic	65.1 (61.9–68.1)	61.3 (57.5–65.0)	72.4 (66.6–77.5)
Hispanic	56.1 (53.8–58.5)	50.6 (47.9–53.3)	68.5 (63.7–72.9)
**Education**			
Less than high school graduate	53.0 (51.2–54.9)	46.8 (44.6–49.0)	65.5 (62.2–68.6)
High school graduate/GED	65.7 (64.8–66.5)	59.8 (58.7–60.9)	76.7 (75.6–77.8)
Some college/technical school	71.4 (70.6–72.2)	66.0 (64.8–67.1)	81.1 (80.0–82.2)
College graduate	75.6 (74.9–76.2)	70.7 (69.8–71.6)	85.0 (84.2–85.8)
**Annual household income ($)**			
<15,000	58.0 (56.3–59.8)	53.4 (51.3–55.4)	66.9 (63.7–70.0)
15,000–34,999	62.2 (61.1–63.2)	54.5 (53.1–56.0)	75.2 (73.9–76.5)
35,000–49,999	67.5 (65.9–69.0)	60.1 (57.9–62.3)	80.3 (78.7–81.8)
50,000–74,999	72.6 (71.4–73.7)	66.2 (64.5–67.8)	83.8 (82.6–85.0)
≥75,000	76.1 (75.4–76.8)	70.8 (69.9–71.8)	87.1 (86.2–88.0)
**Residence location**			
Metropolitan^¶^	72.8 (71.6–73.9)	68.1 (66.5–69.7)	80.2 (78.8–81.4)
Nonmetropolitan	69.4 (68.4–70.4)	64.4 (63.0–65.8)	77.6 (76.3–78.7)
**Health insurance status**			
Yes	71.2 (70.8–71.7)	66.7 (66.0–67.3)	79.7 (79.0–80.3)
No	40.1 (37.3–43.0)	32.6 (30.5–34.7)	55.7 (48.7–62.5)
**Regular health care provider status**			
Yes	72.9 (72.4–73.4)	68.1 (67.5–68.8)	81.6 (81.0–82.3)
No	36.1 (34.6–37.7)	32.7 (31.1–34.4)	45.6 (42.2–49.0)

**Abbreviations:** AI/AN = American Indian/Alaska Native; CI = confidence interval; GED = general educational development certificate.

* Blood stool test within the past 1 year, sigmoidoscopy within the past 5 years, and/or colonoscopy within the past 10 years.

^†^ Data were weighted to the age, sex, and racial/ethnic distribution of each state’s adult population using intercensal estimates and were age-standardized to the 2018 BRFSS population.

^§^ All reported data significantly different (p<0.005) by age group (50–64 years compared with 65–75 years).

^¶^ Metropolitan is defined as in the center city of a metropolitan statistical area (MSA) or outside the center city of an MSA but inside the county containing the center city. Nonmetropolitan is defined as inside a suburban county of the MSA, in an MSA that has no center city, or not in an MSA.

**FIGURE:**
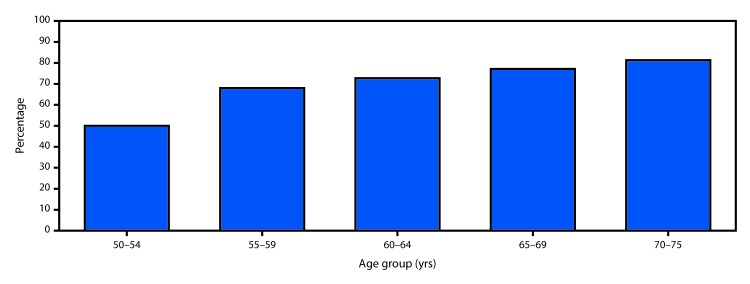
Percentage of respondents aged 50–75 years who reported being up to date* with colorectal cancer screening, by age — Behavioral Risk Factor Surveillance System (BRFSS), United States, 2018^†^^,§^ * Blood stool test within the past 1 year, sigmoidoscopy within the past 5 years, and/or colonoscopy within the past 10 years. ^†^ Data were weighted to the age, sex, and racial/ethnic distribution of each state’s adult population using intercensal estimates and age-standardized to the 2018 BRFSS population. ^§^ Test for trend is significantly different (p<0.005).

Among younger respondents (those aged 50–64 years), reported CRC screening prevalence was lowest among those without a regular health care provider (32.7%) and those without health insurance (32.6%) and highest among those reporting an annual household income of ≥$75,000 (70.8%) and college graduates (70.7%) (Table 1). In this age group, the percentage of respondents who were up to date with CRC screening was higher among women (65.4%), those with health insurance (66.7%), those with a regular health care provider (68.1%), and those living in metropolitan areas (68.1%) than it was among men (61.1%), those without health insurance (32.6%), those without a regular health care provider (32.7%), and those living in non-metropolitan areas (64.4%). As education level and annual household income increased, the percentage of respondents who were up to date with CRC testing also increased.

Among older respondents (aged 65–75 years), reported screening prevalence was lowest among those without a regular health care provider (45.6%) and highest among those who reported annual household income ≥$75,000 (87.1%). Similar to respondents aged 50–64 years, the percentage up to date with screening was higher among women, those with health insurance, those with a health care provider, and those living in metropolitan areas and increased with increasing education and annual household income levels. Overall, 81% of respondents aged 50–64 years and 94% of those aged 65–75 years who had never been screened reported having health insurance.

Among states, Massachusetts had the highest percentage of all adults aged 50–75 years and those aged 50–64 years who were up to date with CRC screening (76.5% and 72.1%, respectively), whereas Wyoming had the lowest percentage (57.8% and 51.5%, respectively) (Table 2). Among adults aged 65–75 years, Rhode Island had the highest percentage who were up to date (84.9%) and Wyoming had the lowest (68.5%). The percentage of adults aged 50–64 years who were up to date was ≥70.5% in four states and <60% in 11 states. In contrast, among adults aged 65–75 years the percentage who were up to date exceeded 70.5% in 49 states and the District of Columbia.

**TABLE 2:** Percentage of respondents aged 50–75 years who reported being up to date* with colorectal cancer screening, by age group and by state — Behavioral Risk Factor Surveillance System (BRFSS), United States, 2018^†^

State	Age group (yrs)		
	Total (50–75)	50–64	65–75
	% (95% CI)	% (95% CI)	% (95% CI)
**United States**	**68.8 (68.3–69.3)**	**63.3 (62.7–63.9)**	**79.2 (78.5–79.8)**
Alabama	69.5 (67.5–71.5)	64.0 (61.2–66.7)	79.3 (76.4–81.9)
Alaska	60.2 (56.6–63.7)	53.3 (48.6–58.0)	73.1 (68.1–77.6)
Arizona	65.2 (62.7–67.7)	59.6 (56.2–62.9)	76.4 (73.4–79.2)
Arkansas	65.5 (62.9–67.9)	58.8 (55.3–62.1)	77.7 (74.8–80.4)
California	70.9 (69.0–72.7)	64.8 (62.4–67.1)	82.4 (79.5–84.9)
Colorado	68.3 (66.6–70.0)	63.3 (61.1–65.6)	77.3 (75.0–79.5)
Connecticut	74.3 (72.8–75.8)	71.2 (69.2–73.1)	80.6 (78.3–82.7)
Delaware	72.2 (70.0–74.4)	67.5 (64.5–70.4)	80.5 (77.4–83.3)
District of Columbia	72.4 (70.0–74.7)	68.7 (65.4–71.8)	79.9 (76.6–82.8)
Florida	69.0 (66.8–71.2)	61.6 (58.5–64.6)	82.9 (80.2–85.3)
Georgia	68.1 (66.3–69.8)	61.7 (59.3–64.0)	80.8 (78.3–83.0)
Hawaii	74.1 (72.1–76.0)	70.2 (67.5–72.7)	81.0 (78.1–83.6)
Idaho	66.1 (63.0–69.0)	60.3 (56.0–64.3)	75.8 (72.2–79.2)
Illinois	66.7 (64.4–69.0)	62.6 (59.6–65.5)	74.8 (71.2–78.0)
Indiana	67.4 (65.5–69.2)	62.2 (59.7–64.5)	77.3 (74.8–79.7)
Iowa	70.9 (69.4–72.4)	66.5 (64.5–68.5)	78.2 (76.0–80.3)
Kansas	66.8 (65.2–68.3)	61.6 (59.5–63.6)	76.1 (74.0–78.1)
Kentucky	69.3 (66.9–71.6)	64.0 (60.8–67.0)	78.6 (75.2–81.7)
Louisiana	69.0 (66.4–71.5)	64.4 (61.0–67.6)	77.3 (73.3–80.9)
Maine	74.7 (73.1–76.3)	70.4 (68.0–72.6)	81.9 (79.9–83.8)
Maryland	72.1 (70.7–73.4)	67.7 (65.9–69.5)	81.3 (79.5–83.0)
Massachusetts	76.5 (74.5–78.4)	72.1 (69.5–74.6)	84.6 (81.8–87.0)
Michigan	73.8 (72.2–75.4)	69.3 (67.2–71.4)	81.4 (78.9–83.7)
Minnesota	73.3 (72.1–74.4)	68.8 (67.2–70.3)	81.3 (79.5–83.0)
Mississippi	62.3 (60.0–64.6)	55.2 (52.1–58.1)	75.6 (72.4–78.5)
Missouri	69.4 (67.1–71.6)	63.7 (60.6–66.7)	79.8 (77.0–82.4)
Montana	63.3 (60.8–65.8)	56.3 (52.9–59.6)	74.7 (71.1–78.0)
Nebraska	68.2 (66.6–69.8)	63.1 (60.9–65.3)	76.5 (74.4–78.5)
Nevada	60.4 (56.5–64.3)	53.8 (48.9–58.6)	74.2 (68.4–79.3)
New Hampshire	74.8 (72.8–76.6)	71.1 (68.5–73.7)	80.9 (78.3–83.3)
New Jersey	67.4 (63.3–71.3)	59.6 (54.2–64.8)	82.6 (77.0–87.1)
New Mexico	63.9 (61.6–66.2)	56.9 (53.8–59.8)	76.0 (73.1–78.8)
New York	69.5 (68.1–70.9)	64.9 (63.1–66.7)	78.2 (75.8–80.4)
North Carolina	70.9 (68.3–73.4)	64.9 (61.5–68.2)	81.7 (77.8–85.1)
North Dakota	66.9 (64.7–69.0)	61.7 (58.8–64.6)	76.3 (73.3–79.0)
Ohio	66.7 (65.1–68.4)	61.4 (59.2–63.6)	76.3 (74.2–78.4)
Oklahoma	62.0 (59.7–64.4)	54.9 (51.7–58.0)	75.2 (72.0–78.1)
Oregon	71.4 (69.1–73.6)	66.6 (63.5–69.6)	80.1 (76.8–83.0)
Pennsylvania	71.4 (69.1–73.5)	66.9 (64.1–69.7)	78.6 (75.4–81.5)
Rhode Island	75.7 (73.5–77.7)	71.0 (68.1–73.8)	84.9 (82.1–87.4)
South Carolina	69.6 (68.0–71.2)	63.5 (61.3–65.7)	80.5 (78.5–82.3)
South Dakota	68.1 (65.2–70.8)	63.8 (60.0–67.4)	76.0 (71.8–79.7)
Tennessee	68.3 (65.7–70.7)	61.5 (58.0–64.8)	81.2 (77.9–84.1)
Texas	59.6 (56.2–63.0)	54.0 (50.0–58.0)	71.6 (65.8–76.8)
Utah	69.8 (68.0–71.5)	64.2 (61.8–66.5)	80.1 (77.6–82.3)
Vermont	71.2 (69.1–73.1)	66.6 (64.1–69.1)	77.5 (74.5–80.2)
Virginia	69.3 (67.5–71.1)	63.8 (61.4–66.1)	80.0 (77.5–82.2)
Washington	70.7 (69.1–72.1)	65.7 (63.6–67.7)	79.3 (77.3–81.2)
West Virginia	67.2 (65.1–69.3)	62.2 (59.3–65.0)	76.6 (73.7–79.4)
Wisconsin	74.8 (72.4–77.1)	70.0 (66.9–73.0)	82.9 (79.4–85.9)
Wyoming	57.8 (55.4–60.1)	51.5 (48.3–54.6)	68.5 (65.3–71.7)

**Abbreviation:** CI = confidence interval.

* Blood stool test within the past 1 year, sigmoidoscopy within the past 5 years, and/or colonoscopy within the past 10 years.

^†^ Data were weighted to the age, sex, and racial/ethnic distribution of each state’s adult population using intercensal estimates and were age-standardized to the 2018 BRFSS population.

## Discussion

An estimated 68.8% of adults aged 50–75 years were up to date with CRC screening in 2018; however, screening prevalence among adults aged 50–64 years was 15.9 percentage points lower than that among persons aged 65–75 years. The percentage up to date with CRC screening varied widely across subgroups, with a 54.5 percentage-point difference between the subgroups with the highest (persons aged 65–75 years with reported annual household income ≥$75,000) and the lowest (persons aged 50–64 years without health insurance) screening prevalence. Up to date CRC screening status increased with increasing age, suggesting that many eligible adults are not receiving important screening that can prevent or detect CRC early, when treatment is more effective.

CRC screening has increased steadily among adults over the past twenty years, but screening prevalence has been consistently higher among those aged 65–75 years than among those aged 50–64 years (*7*). Visits to a primary care provider have been associated with participation in CRC screening (*8*–*10*). In one study, among Medicaid enrollees who had reached age 50 years within the study time frame, 75% had at least one primary care visit within 1 year, but only 17% were screened for CRC during that year. The percentage who initiated screening increased as the number of primary care visits within the previous year increased (*9*). Having a primary care visit at age 49 years was associated with higher CRC screening initiation at age 50 years, but only 69% of patients saw a provider at age 49 years (*11*). Modeling studies have estimated that initiating screening at age 50 years results in larger decreases in population CRC incidence and mortality than when screening is started at age 55 years, suggesting that delayed or slow uptake of CRC screening might diminish the beneficial effect of screening on the population (*12*).

Although lack of health insurance has been strongly associated with low CRC screening prevalence (*7*), the majority of persons in this study who had never been screened reported having health insurance. Other patient barriers to CRC screening include lack of a provider recommendation, being offered colonoscopy only instead of a choice of tests, lack of awareness of the need to be screened, fear, expense, competing priorities, inability to take time off work if referred for a colonoscopy, and the perceived undesirable nature of screening tests (e.g., sampling and storing fecal matter for stool tests or completing a bowel preparation for colonoscopy) (*13*–*16*). Other factors positively associated with CRC screening include use of other preventive services such as cholesterol testing, receiving influenza vaccination, and mammography or cervical cancer screening. Factors negatively associated with CRC screening include provider workload and increasing distance to facilities that perform colonoscopy. Patient comorbid disease might be positively associated with CRC screening because these patients might see their health care provider more frequently, thus increasing the number of opportunities to offer screening, or negatively associated as patients with multiple comorbid diseases might be sicker and unable to participate in screening (*10*,*11*,*17*,*18*). Less is known about how these factors vary and consequently affect CRC screening by age.

There was substantial state variation in the percentage of adults aged 50–75 years who reported being up to date with CRC screening. Given that adults aged 50–64 years accounted for 60%–70% of the population of adults aged 50–75 years in each state, in general, states with the highest reported CRC screening prevalence for this age group also had the highest overall CRC screening prevalence. Variations in the percentage of the population without health insurance, who are racial/ethnic minorities, or who live in rural or frontier areas, as well as the availability of providers who perform colonoscopy and the number of primary care providers per capita, might also contribute to differences in CRC screening by state.

The findings in this report are subject to at least three limitations. First, CRC screening prevalence might be overestimated because BRFSS does not specify whether tests were done for screening or diagnostic purposes. Second, data are self-reported and not validated by medical record review. Third, response rates were low (49.9%), although the BRFSS weighting procedure accounts for nonresponse, and 7.2% of all respondents did not answer all of the questions and were excluded from the analysis.

CRC screening is a grade A recommendation from the U.S. Preventive Services Task Force, meaning that there is strong evidence it effectively decreases CRC incidence and mortality. A microsimulation modeling study found that increasing CRC screening prevalence to 80% had the potential to decrease CRC incidence and mortality by 22% and 33% respectively by 2030 (*19*). This would result in 277,000 new cases averted and 203,000 deaths prevented by 2030. These results assume that participants start screening at age 50 years and continue periodic screening as recommended through age 75 years.

Whereas 68.8% of the U.S. population aged 50–75 years reports being up to date with CRC screening, screening prevalence is lower among younger adults, especially those aged 50–54 years. To achieve further increases in CRC screening to maximize benefit, specific efforts to increase screening in persons aged 50–64 years are needed. Partnerships between public health and health care systems to implement evidence-based interventions such as those described in The Community Guide (*20*) (e.g., provider reminders, patient reminders, provider assessment and feedback, and reduction of structural barriers) can increase CRC screening even in hard-to-reach populations, as demonstrated by CDC’s Colorectal Cancer Control Program (CRCCP) (https://www.cdc.gov/cancer/crccp/about.htm). The CRCCP funds states, tribes, and universities to partner with primary care clinics to implement evidence-based interventions to increase CRC screening. Over 3 years, recipients partnered with approximately 600 clinics reaching approximately 1 million adults aged 50–75 years. Most clinics were Federally Qualified Health Centers, which provide health care in underserved areas. Implementation of evidence-based interventions resulted in an average 10 percentage-point increase in screening prevalence in participating clinics. Additional efforts might include educating adults about the benefit of screening well before age 50 years so that screening can start at age 50 years, providing education about insurance coverage for preventive services, providing clear communication about test options, and conducting research to identify and understand barriers and facilitators to CRC screening specific to this younger age group to inform effective interventions to increase screening.

**Summary**

**What is already known about this topic?**

Screening for colorectal cancer (CRC), the second leading cause of cancer death among cancers affecting men and women, reduces incidence and mortality. The percentage of persons who report being up to date with CRC screening has increased, but not equally among all populations.

**What is added by this report?**

In 2018, 68.8% of adults were up to date with CRC screening test use, but screening prevalence was 15.9 percentage points lower among those aged 50–64 years than among those aged 65–75 years.

**What are the implications for public health practice?**

Specific population-based efforts to increase CRC screening are needed so that screening might start at age 50 years and continue as recommended through age 75 years for maximum benefit.

## Footnotes

* The following questions were asked as part of the Colorectal Cancer Screening module: “A blood stool test is a test that may use a special kit at home to determine whether the stool contains blood. Have you ever had this test using a home kit?,” “How long has it been since you had your last blood stool test using a home kit?,” “Sigmoidoscopy and colonoscopy are exams in which a tube is inserted in the rectum to view the colon for signs of cancer or other health problems. Have you ever had either of these exams?,” “For a sigmoidoscopy, a flexible tube is inserted into the rectum to look for problems. A colonoscopy is similar, but uses a longer tube, and you are usually given medication through a needle in your arm to make you sleepy and told to have someone else drive you home after the test. Was your most recent exam a sigmoidoscopy or a colonoscopy?,” and “How long has it been since you had your last sigmoidoscopy or colonoscopy?.”

† Current U.S. Preventive Services Task Force recommendations for CRC screening include more detailed test type information than can be fully assessed using the data available in BRFSS. To best match the updated recommendations, “up to date” was defined as the percentage of adults aged 50–75 years who reported having had a blood stool test (FOBT or FIT) within the past year, a sigmoidoscopy within the past 5 years, and/or a colonoscopy within the past 10 years.

## References

[1] US Cancer Statistics Working Group. United States cancer statistics: data visualizations. Atlanta, GA: US Department of Health and Human Services, CDC; 2019. https://www.cdc.gov/cancer/dataviz

[2] Bibbins-Domingo K, Grossman DC, Curry SJ, ; US Preventive Services Task Force. Screening for colorectal cancer: US Preventive Services Task Force recommendation statement. JAMA 2016;315:2564–75. 10.1001/jama.2016.5989 27304597

[3] National Cancer Institute. SEER*Explorer. Bethesda, MD: US Department of Health and Human Services, National Institutes of Health, National Cancer Institute; 2019. https://seer.cancer.gov/explorer/

[4] Edwards BK, Ward E, Kohler BA, Annual report to the nation on the status of cancer, 1975–2006, featuring colorectal cancer trends and impact of interventions (risk factors, screening, and treatment) to reduce future rates. Cancer 2010;116:544–73. 10.1002/cncr.24760 19998273 PMC3619726

[5] US Department of Health and Human Services. Healthy people 2020. Washington, DC: US Department of Health and Human Services, Office of Disease Prevention and Health Promotion; 2020. https://www.healthypeople.gov/2020/leading-health-indicators/2020-lhi-topics/Clinical-Preventive-Services

[6] CDC. Behavioral Risk Factor Surveillance System survey data. Atlanta, GA: US Department of Health and Human Services, CDC; 2018. https://www.cdc.gov/brfss/

[7] Hall IJ, Tangka FKL, Sabatino SA, Thompson TD, Graubard BI, Breen N. Patterns and trends in cancer screening in the United States. Prev Chronic Dis 2018;15:E97. 10.5888/pcd15.170465 30048233 PMC6093265

[8] Halm EA, Beaber EF, McLerran D, Association between primary care visits and colorectal cancer screening outcomes in the era of population health outreach. J Gen Intern Med 2016;31:1190–7. 10.1007/s11606-016-3760-9 27279097 PMC5023609

[9] Mojica CM, Bradley SM, Lind BK, Gu Y, Coronado GD, Davis MM. Initiation of colorectal cancer screening among Medicaid enrollees. Am J Prev Med 2020;58:224–31. 10.1016/j.amepre.2019.09.015 31786031 PMC7359742

[10] Weiss JM, Smith MA, Pickhardt PJ, Predictors of colorectal cancer screening variation among primary-care providers and clinics. Am J Gastroenterol 2013;108:1159–67. 10.1038/ajg.2013.127 23670114 PMC3741068

[11] Wernli KJ, Hubbard RA, Johnson E, Patterns of colorectal cancer screening uptake in newly eligible men and women. Cancer Epidemiol Biomarkers Prev 2014;23:1230–7. 10.1158/1055-9965.EPI-13-1360 24793956 PMC4082473

[12] Knudsen AB, Zauber AG, Rutter CM, Estimation of benefits, burden, and harms of colorectal cancer screening strategies: modeling study for the U.S. Preventive Services Task Force. JAMA 2016;315:2595–609. 10.1001/jama.2016.6828 27305518 PMC5493310

[13] Honein-AbouHaidar GN, Kastner M, Vuong V, Systematic review and meta-study synthesis of qualitative studies evaluation facilitators and barriers to participation in colorectal cancer screening. Cancer Epidemiol Biomarkers Prev 2016;25:907–17. 10.1158/1055-9965.EPI-15-0990 27197277

[14] Muthukrishnan M, Arnold LD, James AS. Patients’ self-reported barriers to colon cancer screening in federally qualified health center settings. Prev Med Rep 2019;15:100896. 10.1016/j.pmedr.2019.100896 31193550 PMC6531912

[15] Nagelhout E, Comarell K, Samadder NJ, Wu YP. Barriers to colorectal cancer screening in a racially diverse population served by a safety-net clinic. J Community Health 2017;42:791–6. 10.1007/s10900-017-0319-6 28168395 PMC5517041

[16] Inadomi JM, Vijan S, Janz NK, Adherence to colorectal cancer screening: a randomized clinical trial of competing strategies. Arch Intern Med 2012;172:575–82. 10.1001/archinternmed.2012.332 22493463 PMC3360917

[17] Nielson CM, Vollmer WM, Petrik AF, Keast EM, Green BB, Coronado GD. Factors affecting adherence in a pragmatic trial of annual fecal immunochemical testing for colorectal cancer. J Gen Intern Med 2019;34:978–85. 10.1007/s11606-018-4820-0 30684199 PMC6544723

[18] Liu BY, O’Malley J, Mori M, The association of type and number of chronic diseases with breast, cervical, and colorectal cancer screening. J Am Board Fam Med 2014;27:669–81. 10.3122/jabfm.2014.05.140005 25201936 PMC4273642

[19] Meester RG, Doubeni CA, Zauber AG, Public health impact of achieving 80% colorectal cancer screening rates in the United States by 2018. Cancer 2015;121:2281–5. 10.1002/cncr.29336 25763558 PMC4567966

[20] US Department of Health and Human Services. The Community Guide. Atlanta, GA: US Department of Health and Human Services; 2020. https://www.thecommunityguide.org/

